# Oedipism and Self-Amputation in a Schizoaffective, Depressed Type Patient: To Heal or Feel Pain?

**DOI:** 10.7759/cureus.17515

**Published:** 2021-08-28

**Authors:** Sukhjeet Sangha, Khushbu Shah, Ganeya Gajaram, Vivek Prasad

**Affiliations:** 1 Psychiatry, Interfaith Medical Center, Brooklyn, USA; 2 Psychiatry, Richmond University Medical Center, Staten Island, USA; 3 Psychiatry, Allina Hospitals, Owatonna, USA; 4 Psychiatry, Mayo Clinic Health System - Locums, Rochester, USA

**Keywords:** depression in medical disease, schizoaffective, major self-mutilation, oedipism, mental, psychiatry and mental health

## Abstract

Major self-mutilation, defined as self-inflicted physical harm without suicidal intent, can be a catastrophic complication of schizoaffective disorder. Oedipism and self-amputation are two sequelae seen in schizoaffective patients. Oedipism is a type of self-mutilation where an individual inflicts an ocular injury to oneself, often leading to blindness. Self-amputation, another complication seen in those with schizoaffective disorder, is defined as the act of deliberately removing healthy limbs. This case report discusses a 39-year-old Ukrainian-American male with a history of schizoaffective disorder who displayed both oedipism and self-amputation behavior of varying extremities. The patient’s plan of care was established once an extensive history was obtained and medical records were consolidated. This report contributes to the literature on rare cases of oedipism and self-amputation in patients diagnosed with schizoaffective disorder, depressed type.

## Introduction

Intentional self-injurious or self-mutilating behavior is a perplexing clinical phenomenon, which is defined by Walsh and Rosen as “deliberate, non-life-threatening, self-effected bodily harm or disfigurement of a socially unacceptable nature” [1]. The more common forms of self-mutilation include cutting or burning of the arms or legs, with more extreme behaviors involving inoculation of the skin, eye enucleation, mutilation of the nose, tongue, or genitals, auto-cannibalism, and self-inflicted castration [1]. Self‐enucleation, also referred to as oedipism, draws reference from the Greek character in Sophocles' classical tragedy who gouged his eyes due to a sense of guilt from an incestuous relationship with his mother [2,3]. Though it is rarely seen in clinical practice, over 50 cases of self-enucleation have been published in English journals, with the first case being reported in 1846 [4]. It can be seen as a manifestation of various psychiatric disorders [5,6]. Schizoaffective disorder is a mental health disorder in which the patient has an abnormal perception of reality along with a depressed mood [7]. These patients can experience hallucinations, delusions, and disordered thinking and behavior that can compromise daily functioning [8].

In rare cases, patients may exhibit acts of major self-mutilation, inflicting bodily harm to oneself without suicidal intent, as was seen in 11.3% of patients in one study [5]. Oedipism was formerly understood to be linked to hypersexuality and mythological and/or hyper-religious delusions [6]. However, based on more recently reported cases, the intent leading up to this act varies, although it is frequently linked to psychotic illnesses [9]. Self-injurious behavior is commonly known to occur in untreated psychosis, predominantly schizophrenia, or as the result of substance use-induced intoxication, such as alcohol, cannabis, hallucinogens like lysergic acid diethylamide and phencyclidine, and stimulants including amphetamine and cocaine [3]. Additional causes include severe obsessive-compulsive disorder, mood disorders with psychotic features, factitious disorder and malingering, and severe character pathology [3]. Interestingly, there have also been reports of auto-enucleation in organic illnesses like neurosyphilis, Lesch‐Nyhan syndrome, Down syndrome, structural brain lesions, and postictal psychosis [3]. Self-amputation, another type of self-mutilation, is the act of deliberately cutting off healthy limbs. Self-mutilation in patients with psychiatric conditions often involves the genitalia [10].

In this report, we present the case of a 39-year-old white Ukrainian male who had exhibited several episodes of oedipism and self-mutilation.

## Case presentation

A 39-year-old white Ukrainian-American male lawyer from Kyiv, Ukraine, presented with a chief complaint, which he stated as follows: “I feel original and it empowers me.” He was admitted to the Coney Island Hospital due to a left-arm laceration that he had inflicted upon himself in an attempt to amputate his arm with an electric saw. He had felt the pain to be unbearable, and hence the patient had called an ambulance. A mental status examination was conducted, and it yielded the following findings. The patient’s general appearance at the time of admission was mildly anxious and guarded, and he looked older than his age due to frontal balding. He described his mood as “okay” with a coherent linear goal-directed thought process and content. At that time, the patient presented as guarded, depressed, exhibited poor eye contact, and showed signs of anhedonia such as social withdrawal and negative feelings towards himself. He denied any suicidal or homicidal ideation at the time, and the remainder of the mental status examination was unremarkable.

The patient stated that his recent attempt to inflict self-harm had been due to his feeling lonely, hopeless, and depressed secondary to a diagnosis of the human immunodeficiency virus (HIV). He indicated that he drank alcohol to help him cope. The patient appeared uneasy when discussing his HIV diagnosis. It was determined that the incident had occurred in the context of severe major depressive disorder with psychotic features and under the influence of alcohol.

His medications upon admission, listed in Table 1, included the following drugs: clozapine, lithium carbonate, citalopram, dolutegravir, emtricitabine, tenofovir alafenamide, docusate sodium, bisacodyl, haloperidol, and lorazepam.

**Table 1:** Patient’s medications upon admission PO: by mouth (orally); PRN: Pro re nata (prescription is taken as needed)

Medications on admission	Indication	Dosage
Clozapine	Psychosis	300 mg PO bedtime and 100 mg PO daily
Lithium carbonate	Suicidal thinking	600 mg PO twice daily
Citalopram	Depression	20 mg PO daily
Dolutegravir	HIV	50 mg PO daily
Emtricitabine tenofovir alafenamide	HIV	200/25 mg PO daily
Docusate sodium	Constipation	200 mg PO daily
Bisacodyl	Constipation	10 mg PO bedtime
Haloperidol	Agitation	5 mg PO PRN
Lorazepam	Anxiety	2 mg PO PRN

After 11 weeks of treatment at the Coney Island Hospital, he was transferred to the South Beach Psychiatric Center for further stabilization. 

Past medical and psychiatric history

The patient had a psychiatric history of schizoaffective disorder, medical history of HIV and hepatitis C (treated during 2008-09), hypothyroidism, right-eye blindness, and below-knee left amputation in 2016 secondary to self-mutilation. His history was remarkable for multiple major self-mutilating behaviors. This had begun in 2014, during a visit to the Netherlands, where he had exposed himself to freezing temperatures that had resulted in frostbite, subsequently leading to the amputation of his left fingers. Within the same year, he had traveled back to Ukraine and had stabbed himself in the right eye with a knife, which had resulted in permanent blindness. During his stay in Ukraine, he had committed two more acts of major self-mutilation; in 2016, he had put his left leg on a train track, which had resulted in a below-knee amputation, and later in 2018, he had dragged his right leg outside a moving tram-train, which had led to the loss of his phalanges. Later that year, he had moved back to the United States, at which point he had attempted to self-amputate his leg by laying on a train track. However, he had been unsuccessful due to local law enforcement intervention. His last attempt at self-mutilation before he had been indefinitely hospitalized had been in December 2018, when he had purchased an electric saw with the intention to amputate his left arm. He had been unsuccessful and called the ambulance due to the pain.

**Figure 1:**
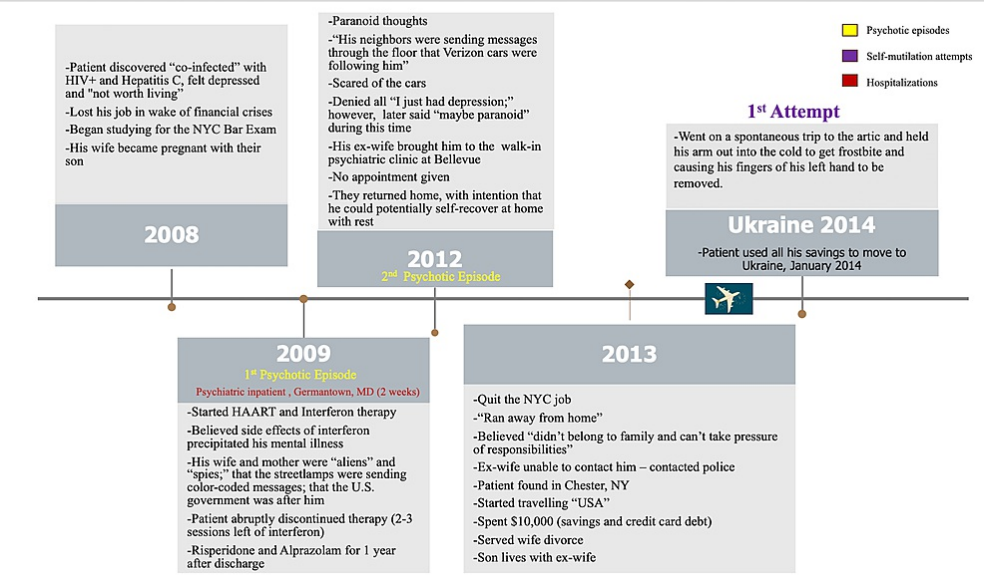
Timeline of patient's self-mutilating and medical history

**Figure 2:**
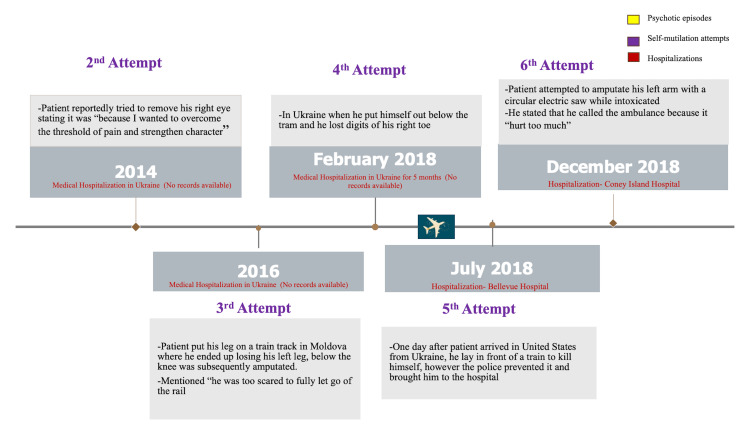
Timeline of patient's self-mutilating and medical history (continued)

The patient had a history of two documented psychotic breakdowns; one in 2009 and the other in 2012. His delusions included paranoid auditory and visual hallucinations with Capgras delusions, which is characterized by a belief in which a familiar person or object is replaced by an imposter. He believed his family members were aliens, that streetlamps were communicating with him, and he was being followed by the government. Upon the patient’s transfer to South Beach Psychiatric Center, his past hospitalization history was revealed, which is summarized in Figure 1 and Figure 2. It was then discovered that he had undergone multiple hospitalizations: Germantown, MD (inpatient, two weeks in 2009), Ukraine (February 2018-July 2018), Bellevue Hospital Center in Brooklyn, NY (inpatient, July 2018-September 2018), Kings County Hospital (partial hospitalization program, September 2018-November 2018) (Table 2).

**Table 2:** Patient's extensive history of hospitalizations D/C: discontinued; D/O: disorder

Period	Cause	Hospital	Length of stay	Diagnosis	Medications
July 2009	First psychotic episode	Psychiatry inpatient, Germantown, MD	2 weeks	Delusional disorder	Risperidone and alprazolam
February 2018–July 24, 2018	Suicidal attempt	Hospital, Ukraine	5–6 months	Depression with psychotic features and delusional D/O	Escitalopram, aripiprazole, risperidone, trazodone, and citalopram
July 25, 2018–September 5, 2018	Suicidal attempt	Bellevue Hospital, NYC	1 month	Schizophrenia	8/6/18 – D/C – aripiprazole started: clozapine and lithium cross-titrated with olanzapine (discontinued later)
September 6, 2018–October 18, 2018	Partial hospitalization program	Kings County Hospital	6 weeks, outpatient	Schizophrenia	Clozapine and lithium (titrated clozapine)
October 19, 2018–December 30, 2018	Intensive outpatient therapy program	Kings County Hospital	12 weeks, outpatient	Schizophrenia	Clozapine and lithium
December 18, 2018–February 27, 2019	Suicidal attempt	Coney Island Hospital	Inpatient	Schizoaffective disorder, depressed type	Clozapine and lithium
February 27, 2019–present	Transfer	South Beach Psychiatric Center	Inpatient	Schizoaffective disorder, depressed type	Clozapine and lithium started; naltrexone

Ultimately, during the hospitalization at Coney Island, he was diagnosed with schizoaffective disorder, depressed type, and he continued to receive clozapine and lithium. He was then transferred to the South Beach Psychiatric Center. 

Social history

The patient described his childhood as a happy one; he said had grown up in a supportive home and denied any history of sexual, physical, or emotional trauma. He reported having been raised Roman Catholic but had not practiced his religion and nor had he undergone any significant religious or spiritual experiences during his adult life. He reported that studying philosophy was the “closest thing to spirituality” in his life. His father had died in 2001, at which time he had left for London to pursue a master’s degree in Law, after which he had gone to Columbia in 2004 to attend law school.

The patient stated that he had worked as a clerk “reviewing documents” from 2005 to 2013, indicating that he could not find a job as a lawyer due to his poor English language skills. He denied ever practicing as a lawyer and admitted to being unemployed since 2013. He endorsed non-compliance with his work schedule and recurrent problems with his manager, which had subsequently resulted in the termination of his employment. He stated that it was during that time that his depressive symptoms had begun to worsen.

He had gotten divorced in 2013 and continued to have an estranged relationship with his ex-wife. It was noted the ex-wife continuously interjected herself into the patient’s healthcare with weekly visits and 10-15 weekly phone calls. The patient expressed his resentment towards her; she refused his visitation rights with his 10-year-old son, whom he had not seen since the age of two.

Collateral information from the patient's mother

As per the patient’s mother, she had not observed any self-harming or suicidal behavior in the patient as a child. She endorsed that he had contracted HIV and hepatitis C during his teenage years, from sharing needles and heroin use at the age of 18. She stated that his older brother felt “embarrassed of his diagnosis” and did not keep in touch with him. She reported a series of bizarre behaviors beginning in 2014; he had exhausted all his savings and flown back to his home country, Ukraine, without informing his family and had appeared at his mother’s house in Kyiv before leaving again to “travel the world in 2014.” She observed that he had looked like a “robot” with “glassy eyes.” She stated that while in Ukraine, he had sporadically disappeared into the wilderness and forests without telling her. She endorsed weekly contact whereby he would call her to say, “I am OK. Bye. Talk to you in a week.” Four years later, in February of 2018, he had called her saying, “I am not in a good state, pick me up.” She described his appearance at that time as “very yellow/jaundiced” and he had been missing his left leg below the knee. This had been the first time that she had been made aware of his physical changes, and she had scheduled a psychiatric appointment for him. She had found him a hotel to stay in that night; however, later in the evening, she had found herself on the other end of a phone call with the police who had stated that they had pulled him out from beneath the tram that he had jumped in front of, resulting in the amputation of his right toes. The patient endorsed that this had been a suicide attempt and he had drunk vodka heavily before the attempt to ease the pain.

Assessment

At the South Beach Psychiatric Center, apart from the aforementioned mental status examination findings, the patient initially denied having a significant substance use history, despite his blood alcohol level being 114 upon admission (the equivalent of roughly five to six 12-oz bottles of beer). The patient reported feeling chronically depressed since 2008 (when he had been diagnosed with HIV and hepatitis C). He stated the discovery had made him feel “hopeless” and not worth living.

When questioned about his major self-mutilation behaviors, he indicated that he had not been attempting suicide; he denied having delusions or hallucinations during those episodes. Instead, he said he had been attempting to understand “his threshold for pain.” He did admit to one prior episode of suicidal attempt and stated that the subsequent episodes of self-harm had been intentional and said that they had been attempts to “just to injure myself.” He reported loneliness as a contributing factor to his depressed mood. He continuously minimized the seriousness of his self-inflicted injuries, stating “it’s just no big deal, I was depressed, so I did it.” According to the patient, he believed that self-mutilation made him feel “empowered.” He stated that he often abused alcohol to numb some of the pain while committing these acts of self-mutilation.

While establishing the patient's care, the biological, psychological, and social factors that potentially affected his condition were analyzed, which is summarized in Table 3.

**Table 3:** Biological, psychological, and social factors that potentially affected the patient’s condition HIV: human immunodeficiency virus; AOT: assisted outpatient treatment

	Biological	Psychological	Social and cultural
Predisposing	Medications (interferon, HIV meds), HIV and hepatitis C diagnosis	Divorce, loneliness, unemployment, and desire to see ex-wife and son	Unstable family dynamic, loneliness, and inadequate family support
Precipitating	Medications (HIV meds), substance use (alcohol), HIV diagnosis, hepatitis C diagnosis, and multiple amputations	Loneliness, unemployment, feelings of being an inadequate lawyer, and schizoid personality	Relocation, financial difficulties, and unstable housing
Perpetuating	Medications (HIV meds), substance use (alcohol), HIV diagnosis, hepatitis C diagnosis, and multiple amputations	Fractured relationship with ex-wife, low self-esteem, and attention-seeking	HIV symptoms and amputations
Protective	Cure of hepatitis C		Access to healthcare, structured environment, AOT, and ex-wife and son

At the unit, the patient exhibited good behavioral control and did not need any pro re nata (PRN) or stat medications. However, he interacted only minimally with staff and peers. He was observed spending a substantial time alone and was reading most of the time.

Diagnosis

The patient had an extensive history of paranoid ideation with perceived threats, Capgras delusions, ideas of reference with attribution of salience to irrelevant stimuli, with auditory and visual hallucinations, and bizarre instances of self-injury. The positive symptoms were episodic in nature and were not elicited during his admission. The negative symptoms included social isolation, alogia, avolition, and flat affect. Due to the patient’s psychotic features, depressed mood, and chronic alcohol dependence, it was concluded that the final diagnosis was schizoaffective disorder, depressed type, and substance abuse disorder related to alcohol. 

Differential diagnoses

The differentials included schizoaffective disorder, depressed type; schizophrenia, undifferentiated; major depressive disorder with psychotic features; personality disorder (cluster B) and schizoid vs schizotypal; body integrity identity disorder; and substance or medication-induced psychotic disorder. Despite the initial focus on depressive symptoms, severe primary psychotic disorder rose high on the differential given the patient’s extensive history of paranoid ideation with perceived threats, Capgras delusions, ideas of reference with attribution of salience to irrelevant stimuli, auditory and visual hallucinations, and bizarre instances of self-injury. A detailed summary of the patient’s differential diagnoses is presented in Table 4. 

**Table 4:** Summary of patient's differential diagnosis

Differential diagnosis	Rationale	
Schizophrenia, undifferentiated		Given the patient's delusions and disorganized behavior, schizophrenia was considered. However, because they were accompanied with depressive symptoms, schizoaffective disorder, depressive type was the more acceptable diagnosis
Major depressive disorder with psychotic features		Although the patient had depressed mood symptoms, psychosis was the predominant symptom
Medication-induced psychotic disorder		Although interferon, a medication used to treat hepatitis C, does have documented side effects of psychosis, the patient’s psychosis persisted despite taking this medication
Substance-induced psychotic disorder		Alcohol-induced psychotic disorder can be a rare complication of chronic alcohol use following an abrupt cessation. However, the patient’s psychosis and depressive symptoms persisted despite abstaining from alcohol or being under the influence

Treatment

The patient was started on naltrexone 50 mg/day to manage his substance abuse (alcohol); this was done to ensure that he maintained his sobriety, which would make him less inclined to self-mutilate since he had a history of such behaviors while intoxicated. Clozapine 400 mg/day was also added to the patient's treatment regimen for his psychosis and to reduce self-harming behaviors [11]. The patient had been previously compliant with lithium, and his lithium level had been 0.7 mEq/L at the time of admission, which was within the therapeutic range [12]. Lithium dose was increased from 1200 mg/day to 1350 mg/day, which was indicated for mood stabilization and also to reduce self-harming behaviors [13]. The patient reported no side effects to any of the medications. His medication regimen is summarized in Table 5. In addition to the psychotropic medications, he was placed in the intensive group and individual therapy. A detailed plan of the patient's care is presented in Table 6. The findings of the patient's dialectical behavioral therapy (DBT) are summarized in Table 7. The patient’s weekly psychotherapy session details are summarized in Table 8.

**Table 5:** Summary of patient's medication regimen

Psychotropic medication	Indication	At admission	During the stay	Side effects
Citalopram	Depression	20 mg/day	Increased to 40 mg daily	Reported no side effects
Clozapine	Psychosis	400 mg/day	400 mg/day	Reported no side effects
Lithium	Mood stabilizer	1200 mg/day	Increased to 1350/day	Reported no side effects
Naltrexone	Self-injurious behavior and craving for alcohol	None	October 2019: 25 mg/day increased after 2 weeks to 50 mg/day	Reported no side effects

**Table 6:** Detailed plan of patient's care CT-R: recovery-oriented cognitive therapy; DBT: dialectical behavior therapy

Patient’s treatment and therapy
Psychotropic medications
Group programming to increase socialization, psychoeducational routines, and learning coping skills
CT-R group for the management of residual symptoms and relapse prevention
Milieu treatment, individual therapy, and both on and off unit programming as well
Social engagement also provided
DBT therapy
Full psychological testing

**Table 7:** Patient's DBT findings DBT: dialectical behavioral therapy

Patient's DBT findings (understanding coping skills)	
Self-identified ineffective coping skills	Extreme self-harm
Self-identified effective coping skills	Reading, challenging philosophy, completing test questions in accounting book, attending events for increased socializing opportunities, and exercising

**Table 8:** Summary of patient's psychotherapy sessions CIH: Coney Island Hospital; BAC: blood alcohol concentration

Patient’s weekly psychotherapy sessions	
Question:	Patient’s answer:
What triggers led to self-harm?	The patient stated he was “lonely, hopeless, and depressed.” However, he denied suicidal ideation, plan, or intent
Why chose self-harm to manage hopelessness rather than healthy coping strategies like reaching out to loved ones, or talking to a mental health professional?	The patient stated, “because self-harm makes me feel more empowered than seeking help from others.” He stated that although now he finds his action illogical, he had acted the way he had done because he had thought that if he took out his eye, it would help him be stronger in the face of his psychiatric symptoms. Also, he admitted to drinking beer/vodka heavily to “ease the pain”
How much alcohol do you or did you consume?	The patient stated he only drank “two pints” prior to his self-harming episode; however, the patient’s alcohol level was noted to be very high upon admission to CIH (i.e., BAC of 114)

Once the patient was stabilized, he was transferred to a community assisted program in Brooklyn, where he would have around-the-clock supervision, aid with medication management, as well as individual and group therapy. He would continue to receive services through HIV/AIDS Services Administration. Additionally, the patient’s family was educated on the ways to manage the patient’s residual symptoms in the community. He is a Social Security Disability recipient and will receive benefits to support his livelihood in the post-discharge environment.

## Discussion

Schizoaffective disorder affects 0.3% of the population; self-mutilating behavior can be a characteristic of schizoaffective disorder [14]. Oedipism is one of the most common types of self-mutilating acts after limbs and genital mutilation [4]. Studies have found that patients who self-mutilate are predominantly males between the ages of 20 and 40 years [4]. This is postulated to be due to men being more impulsive and tending to act out more frequently [4]. The psychoanalytic model explains the acting out as an expression of the dramatization of the processes of struggle against annihilation taking root in the initial depression and reactivated thereafter in oedipism [4]. The risk of oedipism is higher among prisoners, socially isolated individuals, and the unemployed [4]. The acts of self-mutilation often involve fingers; however, there have been a few incidents where patients have used objects like sharp scissors, spoons, and air guns [4]. Our patient was admitted due to an attempt at self-mutilating his left arm with an electric saw. He had blindness in the right eye due to self-mutilation with a knife. His self-mutilating behavior had begun in 2014 at the age of 33 while unemployed. As per his mother, this patient had a history of running away and being isolated for long periods of time.

One case study highlighted oedipism being triggered by delusional ideas of influence, possession, erotomania, and persecution [4]. Sexual themes are found in 30% of cases; some consider the eye as a phallic symbol and self-enucleation as associated with the idea of self-castration especially in the presence of oedipal or homosexual conflicts [4]. In fact, most cases described in the literature of oedipism were due to severe mental disorders, especially psychosis [4]. Though oedipism was initially thought to be related to preoccupations with sins and higher deities, several recently published case reports highlight that this is a universal phenomenon that can be observed in people with different religious and cultural backgrounds [4,15]. our patient was under the influence of alcohol when he attempted to self-amputate his left arm; it was difficult to ascertain whether he was also experiencing delusions or hallucinations that guided this episode of self-mutilation. He did, however, had a history of paranoid ideation with perceived threats, Capgras delusions, ideas of reference with attribution of salience to irrelevant stimuli, with auditory and visual hallucinations; however, it was difficult to determine whether these had in any way caused the previous episodes of self-mutilation and oedipism.

Various theories have been postulated regarding the etiology of self-mutilation [4,16]. Earlier, psychiatrists posited that self-enucleation resulted from oedipal guilt due to patients feeling incestuous desire towards their mothers [4]. More recent theories implicate a psychological function whereby self-mutilation relieves internal tensions and negative emotions [4]. These behaviors could increase the secretion of endorphins or dopamine that are lacking in psychotic patients [4]. This theory is supported by our case as the patient endorsed feeling depressed during the episodes of self-mutilation. Furthermore, it has been well documented that he had been uncomfortable with his diagnosis of HIV. He also endorsed that one previous episode of self-mutilation had been driven by the desire to determine his pain threshold. His reasoning for his oedipism and self-amputation was an associated feeling of empowerment.

In addition to the psychological theories behind self-mutilating behaviors, several biological theories have been proposed as well [4]. Several disorders have been implicated in the occurrence of self-mutilation, such as Lesch-Nyhan syndrome, Prader-Willi syndrome, and Gilles de la Tourette syndrome, which may be caused by a disruption in the serotonergic neurotransmission [4]. Other organic disorders that may present with self-mutilating behaviors include temporal epilepsy, neurosyphilis, frontal lobe encephalomalacia, Down syndrome, and delirium tremens [4]. Additionally, dopaminergic dysfunction, which increases the threshold of pain, may play a role in self-mutilation [4]. In fact, a meta-analysis published in 2015 showed a decrease in pain sensitivity and an increase in the threshold of pain reactivity in schizophrenic patients [4]. This could be related to opioid receptor abnormalities and N-methyl-d-Aspartate (NMDA) receptor and dopamine receptor dysregulations [4]. Moreover, some studies have shown a decrease in activity in the regions involved in the pain process such as the thalamus, insula, hippocampus, somatosensory cortex I and II, prefrontal cortex, lower parietal cortex, and cerebellum [4]. Further testing is required to determine whether the patient had any NMDA, opioid, or dopamine receptor dysregulations as well as any structural abnormalities or abnormal activity levels in the regions involved in pain processing. The patient denied any history of Lesch-Nyhan syndrome, Prader-Willi, Tourette syndrome, epilepsy, syphilis, frontal lobe encephalomalacia, or Down syndrome. He was also not observed to be in alcoholic withdrawal at the time of admission; however, any prior history of delirium tremens is unknown so it cannot be ruled out as a potential etiology of one of his episodes of self-mutilation. His history of schizophrenia could explain the neurobiological basis underpinning his self-mutilating tendencies, as evidenced by his remission with clozapine and lithium.

Besides the neurobiological bases of self-mutilation, several psychiatric disorders have also been identified [4]. In addition to schizophrenia, the literature documents obsessive-compulsive disorder, post-traumatic stress disorder, depression, Munchausen syndrome, and borderline personality disorder [4]. Patients often lack insight into schizophrenia spectrum disorders; however, studies have shown that with continued pharmacologic and therapeutic interventions, patients' insight may significantly increase [17]. Timely interventions can help reduce the risk of future self-mutilating behaviors. Although several other conditions such as schizophrenia, bipolar disorder, and major depressive disorder were considered by way of differential diagnosis, the patient was ultimately diagnosed with schizoaffective disorder, depressed type. His illness had been characterized by uninterrupted psychotic durations along with major depressed mood features, thereby satisfying the Diagnostic and Statistical Manual of Mental Disorders, 5th Edition (DSM-5) criterion for the diagnosis [18].

Though the literature describes sexual themes related to oedipism as a result of homosexuality, there was insufficient information about his sexual orientation and his level of comfort with his sexuality to determine if this may have played a contributory role in his oedipism.

It is difficult to speculate on the driving force that had led this patient to self-mutilation. It could be postulated that because the patient experienced symptoms of psychosis concurrently with the episodes of self-mutilation, the act of self-mutilation may have been carried out with the intent to increase the secretion of endorphins and dopamine, which are typically lacking in psychotic patients [4]. Though the patient endorsed being raised a Catholic, he denied any religious practice. Therefore, a relationship between his religious beliefs and atonement through self-mutilation is not supported in this case.

The use of alcohol or drugs, including LSD, cocaine, cannabis, and amphetamines, has been implicated in the amplification of psychotic episodes leading to self-mutilation [4]. The patient’s blood alcohol concentration at the time of admission after his arm amputation attempt was 114, which satisfies the criterion for legally intoxicated.

## Conclusions

We discussed the case of a patient with a history of schizoaffective disorder, depressed type, with major self-mutilation behavior. The patient’s self-mutilation episodes included oedipism and several self-amputations that had occurred over a period of four years. Self-mutilation in patients with psychiatric conditions often involves the genitalia and not the eyes or extremities as seen in this patient. A patient self-inflicting harm, amputation, and even blindness, is scarcely seen in clinical practice. When reviewing the literature, it is evident that each case is unique: from the etiology of each episode of self-mutilation to the ensuing treatment that must address the respective underlying pathology. The literature documents the heterogeneity of etiologies underpinning episodes of self-harm, especially self-amputation and oedipism. This case report contributes to the currently scarce body of literature on patients exhibiting oedipism, self-amputation, and self-mutilation partly due to an underlying psychiatric condition of schizoaffective disorder. Further studies are required to substantiate or rule out any neurobiological and neuroanatomical basis of the self-mutilating behavior. Also, further psychological analyses are warranted to solidify or rule out the psychological basis of self-mutilation.
